# MiR-210 inhibits NF-κB signaling pathway by targeting DR6 in osteoarthritis

**DOI:** 10.1038/srep12775

**Published:** 2015-08-05

**Authors:** Dawei Zhang, Xiaorui Cao, Jun Li, Guangyue Zhao

**Affiliations:** 1 Department of Orthopedics, Xijing Hospital, Fourth Military Medical University, Xi’an 710032, China

## Abstract

Osteoarthritis (OA) is characterized by degradation of articular cartilage and joint inflammation. MicroRNAs have been proved to play an important role in the regulation of chondrogenesis. Previous study showed that microRNA-210 (miR-210) was probably associated with osteoarthritis, while the function of miR-210 in osteoarthritis still remains unknown. The aim of the present study was to investigate the protective effect of miR-210 on osteoarthritis. In the *in vitro* study, miR-210 level in chondrocytes was decreased after treatment with lipopolysaccharide (LPS). Transfection with miR-210 mimic inhibited LPS-induced pro-inflammatory cytokines production, cell viability reduction and cell apoptosis. Results of luciferase activity assay showed that miR-210 targeted 3′-UTR of death receptor 6 (DR6) to inhibit its expression. MiR-210 mimic and DR6 siRNA transfection inhibited the activation of NF-κB pathway and cell apoptosis of chondrocytes. For the *in vivo* study, OA model was established on rats by anterior cruciate ligament transection (ACLT). MiR-210 expression is reduced in OA rats. MiR-210 over-expressing lentivirus was injected into the OA rats. Cytokines production, and NF-κB and DR6 expression in OA rats was inhibited by miR-210 overexpression. The results demonstrated that miR-210 decreased inflammation in articular cavity in OA rats by targeting DR6 and inhibiting NF-κB signaling pathway.

Osteoarthritis (OA) is one of the most common degenerative joint disease which is characterized by degradation of articular cartilage and joint inflammation^1^. Repair and degradation of articular cartilage are imbalanced in OA^2^. MicroRNAs (miRNAs) are a group of small non-coding RNAs which bind to target mRNAs to interfere the translation process^3^. MiRNAs possess diverse functions, including the regulation of cellular differentiation, proliferation and apoptosis, as well as cancer initiation and progression^4^,^5^. Several miRNAs exhibit tissue- or developmental stage specific expression pattern and associate with diseases such as cancer, heart disease, diabetes and rheumatoid arthritis^6^,^7^,^8^,^9^,^10^. Recently, miRNAs have been proved to play an important role in chondrogenesis and OA^11^. Previous study showed that microRNA-210 (miR-210) was probably associated with OA^12^, while the function of miR-210 still remains unknown.

Since inflammation is a feature of OA, the presence of up-regulated levels of pro-inflammatory cytokines in the synovial fluid (SF) indicates synovitis in early OA^1^. MiR-210 has been shown to be an inhibitor of proinflammatory cytokines production^13^. A recent report showed that miR-210 associates with nuclear factor kappa-B (NF-κB) signaling pathway which is a well conserved, ubiquitous, and pivotal regulator of the immune response, inflammation and cell survival^14^,^15^,^16^,^17^. However, exact mechanism between miR-210 and NF-κB pathway has bot been fully illustrated. Tumor nectosis factor (TNF) receptors are key players in inflammation and immune regulation. Death receptor 6 (DR6), also known as TNF receptor superfamily member 21 (TNFRSF21), has been shown to induce cell apoptosis and activation of NF-κB^18^. Previous study reported that DR6 was up-regulated in OA^19^, thus we hypothesize DR6 might be a molecular mediator between miR-210 and NF-κB pathway.

The aim of this study was to evaluate the role and its mechanism of miR-210 in OA. We found that miR-210 targeted DR6 and inhibited the activation of NF-κB pathway both *in vivo* and *in vitro*. The results indicated that miR-210 might be a medical target fot the treatment of OA.

## Results

### MiR-210 expression was inhibited by LPS in chondrocytes

**Figure 1:**
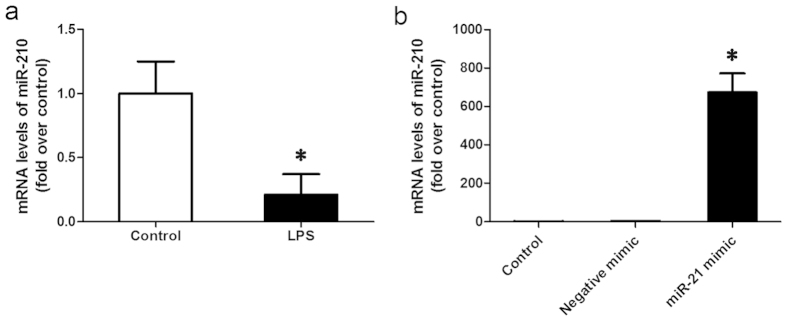
mRNA levels of miR-210 in chondrocytes. (**a**) MiR-210 mRNA level in chondrocytes with LPS/PBS treatment; (**b**) MiR-210 mRNA level in chondrocytes transfected with negative mimic or miR-210 mimic. Data are shown as mean ± SD of three experimental replicates. *P < 0.05 vs. control group.

The isolated chondrocytes were treated with LPS to induce inflammation *in vitro*, the expression level of miR-210 was detected by RT-PCR. As shown in Fig. 1a, the miR-210 level was decreased by 75% in LPS induced cells. To investigate the effect of miR-210 on LPS-induced inflammation in chondrocytes, the isolated chondrocytes were transfected with miR-210 mimic or negative mimic. Expression of miR-210 in chondrocytes was significantly enhanced after transfection with miR-210 mimic (Fig. 1b).

### Pro-inflammatory cytokines production, cell viability reduction and cell apoptosis induced by LPS were alleviated by miR-210 mimic

**Figure 2:**
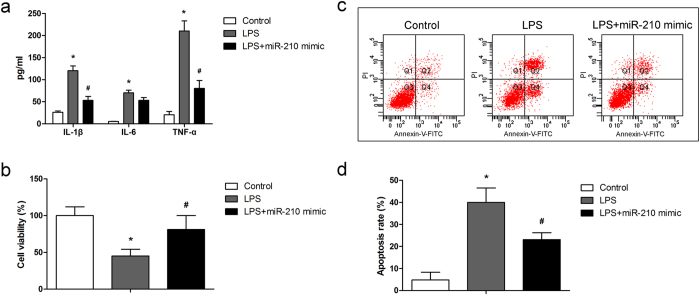
Effect of miR-210 on LPS-induced chondrocytes. (**a**) Production of IL-1β, IL-6 and TNF-α in the cell culture supernatants was determined by ELISA; (**b**) Cell viability was detected by MTT assay; (**c**–**d**) Cell apoptosis was measured by FCM. Data are shown as mean ± SD of three experimental replicates. *P<0.05 vs. control group; ^#^*P *< 0.05 vs. LPS group.

LPS usually induces inflammation and causes cell apoptosis. After incubation with LPS, levels of IL-1β, IL-6 and TNF-α in cell supernatant were detected by ELISA. Results showed that LPS induced secretion of IL-1β, IL-6 and TNF-α (Fig. 2a). The levels of IL-1β and TNF-α were decreased in miR-210 mimic transfected cells (Fig. 2a). The cell viability of chondrocytes was detected by MTT assay. As shown in Fig. 2b, the relative cell viability was decreased by 55% after the treatment of LPS, whereas the cell viability was increased by 32% after miR-210 transfection. Flow cytometry was utilized to quantify LPS-induced chondrocytes apoptosis. As shown in Fig. 2c,d, the percentage of cell apoptosis was increased to 39.1% in LPS-induced cells (*P *< 0.01), and reduced to 25.6% after miR-210 mimic transfection (*P *< 0.05). The results indicated that miR-210 protect the chondrocytes from LPS induced injury.

### MiR-210 targeted DR6 in chondrocytes

**Figure 3:**
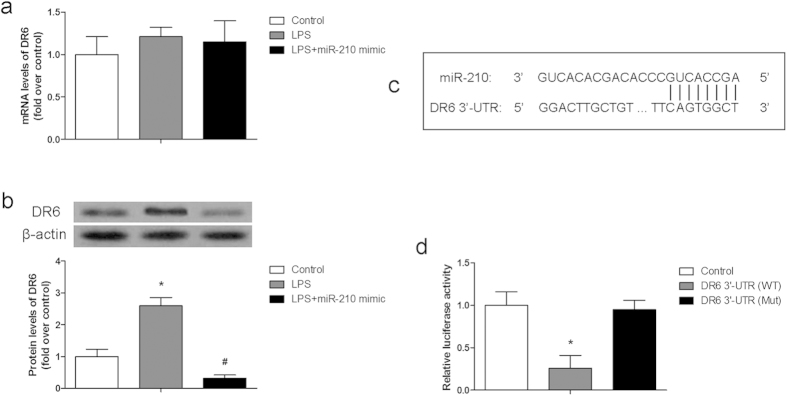
MiR-210 targeted DR6 in chondrocytes. (**a**) mRNA levels of DR6 in chondrocytes with different treatment; (**b**) Protein levels of DR6 in chondrocytes with different treatment; (**c**) prediction result of miR-210 targeting DR6 3′-UTR; (**d**) relative luciferase activity of 293T cells with different treatment. Data are represented as mean ± SD of three experimental replicates. *P < 0.05 vs. control group, ^#^*P *< 0.05 vs. LPS group.

To investigate the molecular mechanism of miR-210, the potential targets of miR-210 was predicted from RegRNA website. DR6 was finally selected as the most likely target of miR-210 during inflammation (Fig. 3c). The mRNA and protein levels of DR6 were detected by RT-PCR and western blotting, respectively. As shown in Fig. 3a,b, protein level of DR6 was increased by LPS and inhibited by miR-210 mimic. However, the change of DR6 mRNA level was not obvious, indicating that the regulation of DR6 expression was posttranscriptional gene silencing (PTGS). To confirm whether miR-210 was targeted at the 3′-UTR of DR6, a relative luciferase activity assay was performed. The relative luciferase activity significantly decreased when the cells were transfected with the wide type of DR6 3′-UTR and miR-210 mimic (Fig. 3d). The results indicated that DR6 was the direct target of miR-210.

### MiR-210 protected chondrocytes through inhibiting NF-κB pathway

**Figure 4:**
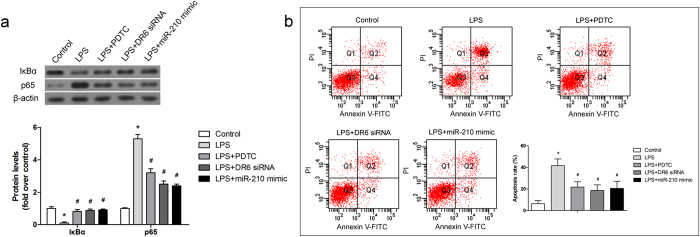
MiR-210 protected chondrocytes through inhibiting NF-κB pathway. (**a**) The expression of IκBα and p65 was detected by western blotting; (**b**) Cell apoptosis was measured by FCM. Data are shown as mean ± SD of three experimental replicates. *P < 0.05 vs. control group; ^#^*P *< 0.05 vs. LPS group.

DR6 has been shown to activate NF-κB and induce cell apoptosis^18^. To investigate whether NF-κB signaling pathway was involved in the protective effect of miR-210, pyrrolidine dithiocarbamic acid (PDTC) which is an inhibitor of NF-κB was used in the study. As shown in Fig. 4a, LPS induced activation of NF-κB signaling pathway with the evidences of p65 induction and IκBα reduction, and the activation was inhibited by PDTC. The regulation of p65 and IκBα caused by LPS was also inhibited by miR-210 mimic and DR6 siRNA. In addition, cell apoptosis caused by LPS was attenuated by PDTC, DR6 siRNA and miR-210 mimic (Fig. 4b), suggesting that miR-210 targeted DR6 and protected chondrocytes from LPS by inhibiting activation of NF-κB signaling pathway.

### MiR-210 inhibited expression of pro-inflammatory cytokines and NF-κB pathway in OA rats

**Figure 5:**
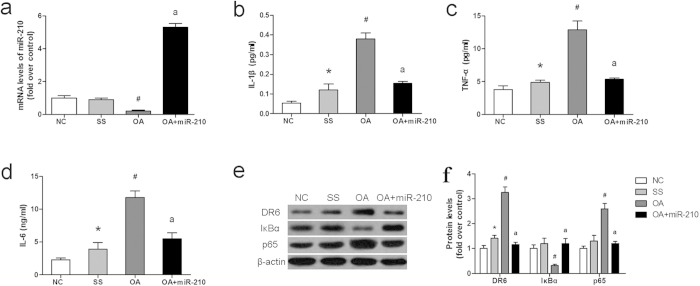
Effect of miR-210 in OA rats. (**a**) The mRNA level of miR-210 in articular cartilages was detected by RT-PCR; (**b**–**d**) inflammatory factors in SF samples were measured by ELISA; (**e**) The expression of DR6, IκBα and p65 in articular cartilages was detected by western blotting; (**f**) the quantitative data for e. Data are shown as mean ± SD of five rats in each group. NC, normal control group; SS, sham surgery group; OA, OA model group; OA+miR-210, OA model with the treatment of miR-210-expressing lentivirus. Data are shown as mean ± SD of three experimental replicates. *P < 0.05 vs. normal control group; ^#^*P *< 0.05 vs. sham surgery group; ^a^*P *< 0.05 vs. OA model group.

To investigate the role of miR-210 *in vivo*, the rat model of OA was built by anterior cruciate ligament transection (ACLT) in the right knees. MiR-210-expressing lentivirus was injected into articular cavity of the OA rats. On the 20 th day after the operation, the rats were sacrificed and the articular cartilages of medial tibial plateau and SF samples were collected for analysis. As shown in Fig. 5a, miR-210 level in the articular cartilages was decreased by 0.8-fold in OA rats and increased by 5.4-fold in lentivirus infected rats. Levels of the inflammatory cytokines in SF samples were measured by ELISA. Levels of IL-1β, IL-6 and TNF-αwere significantly increased by 3, 2.5, 1.5, 1.7, 1.8 and 1.5 fold in OA rats compared with sham surgery group. However, miR-210 overexpression inhibited the production of cytokines in SF samples (Fig. 5b–d). Expression of IκBα and p65 in OA rats were changed compared with sham surgery group (*P *< 0.05), while the effect could be inhibited by miR-210 overexpression (Fig. 5e,f).

## Discussion

OA is a complex, multifactorial inflammatory disease of the whole joint^20^. More than 25 miRNAs have been demonstrated to associate with OA and many are functionally implicated in the pathogenesis of the disease^11^. MiR-140 which is specifically expressed in cartilage regulates cartilage development and homeostasis, and its loss contributes to the development of age-related OA-like changes^21^. It was reported that miR-21 is up-regulated in osteoarthritis patients, and overexpression of miR-21 attenuates the process of chondrogenesis^22^. Previous studies indicated that miR-210 may be associated with OA^12^,^23^. An OA model of rat was built successfully in this study. Results showed that the miR-210 expression in articular cartilage of OA rats and LPS-simulated chondrocytes was much less abundant than that of normal rats, suggesting miR-210 may play an important role in OA.

MiRNAs play an important role in both adaptive and innate immunity^24^. MiR-210 overexpression inhibits TLR4-induced secretion of pro-inflammatory cytokines IL-6 and TNF-α^13^. MiR-210 was also confirmed to be a feedback negative regulator for LPS-induced inflammation^13^. MiR-210 is always overexpressed in tumors and inhibits cell apoptosis to promote cancer progress. It has been shown that miR-210 is also associated with hypoxia pathway and protect cells from hypoxia-induced apoptosis^25^. Overexpression of miR-210 in epithelial ovarian cancer promotes tumor growth via inhibiting cell apoptosis^26^. In this study, transfection of miR-210 mimic exhibited anti-inflammatory and anti-apoptotic effects in LPS-induced chondrocytes. For the *in vivo* assay, miR-210-expressing lentivirus were injected into the OA rats to investigate the role of miR-210. The results also showed that miR-210 possess anti-inflammatory effects.

NF-κB plays a key role in regulating the immune response. Incorrect regulation of NF-κB has been linked to cancer, inflammatory, and improper immune development. It has been reported that several microRNAs have been demonstrated to negatively regulate NF-κB activation and the subsequent production of proinflammatory cytokines^13^. However, a study demonstrated that overexpression of miR-210 under hypoxia was regulated by NF-κB transcriptional factor p50^23^. Besides, transfection of siRNAs of NF-κB also reduces miR-210 expression^27^. The results indicate that miR-210 acts as a feedback regulator of NF-κB pathway. IκBα is a cellular protein which inhibits the NF-κB activation by masking the nuclear localization signals of NF-κB proteins and keeping them sequestered in an inactive state in the cytoplasm. It is a quite important marker of NF-κB activation. In the present study, whether the anti-apoptotic effect of miR-210 was mediated by NF-κB pathway was evaluated. As shown in Fig. 4, miR-210 played a similar role with PDTC to inhibit NF-κB activation by reducing the p65 expression and increasing the IκBα level in LPS treated cells. To further evaluate the molecular mechanism of miR210, its target gene was confirmed. As shown in Fig. 3, DR6 which is an important protein to induce cell apoptosis and activation of NF-κB was confirmed to be the target of miR-210.

In conclusion, the results demonstrate miR-210 expression was decreased both in LPS-induced chondrocytes and OA rats. MiR-210 overexpression exhibited anti-apoptotic and anti-inflammatory effects. Then we found miR-210 targeted 3′-UTR of DR6 to inhibit its expression. MiR-210 mimic and DR6 siRNA inhibit the activation of NF-κB and cell apoptosis of chondrocytes. The results demonstrated that miR-210 alleviated inflammation in articular cavity in OA rats by targeting DR6 and inhibiting NF-κB signaling pathway, suggesting that miR-210 might be a medical target for the treatment of OA.

## Methods

### Animals

Twenty-six female Sprague-Dawley rats (Charles River Laboratories, 8-weeks post-weaned, 210–250 g) were housed in a temperature-controlled room (22–24 °C), with a 12–12 h light-dark cycle. Animals were given free access to chow and water. All procedures in this study were approved by the Animal Care Committee of Fourth Military Medical University, and all the methods were carried out in accordance with the approved guidelines.

### Primary rat chondrocytes isolation and culture

Primary chondrocytes were isolated from six healthy female rats as described previously^28^. Slices of cartilage were stripped from the femoral condyles and tibial plateau. The slices were cut into small pieces and digested in growth media (DMEM supplemented with 10% FCS, 100 μg/ml streptomycin, 2 mmol/l _L_-glutamine, 100 U/ml penicillin, non-essential amino acids (Invitrogen) and 2.5 μg/ml amphotericin containing 1% collagenase type IA (0.5–3.0 FALGPA U/mg, Sigma, Dorset, UK). After incubation at 37 °C for 24 hours, the sample was filtered to remove undigested cartilage and chondrocyte cells were pelleted at 2000 *g* for 5 min before being resuspended in growth media. LPS were used at a final concentration of 100 ng/ml. To evaluate the role of NF-κB pathway during LPS-induced inflammation in this study, cells were pretreated with pyrrolidine dithiocarbamate (PDTC) (10 μmol/l, Sigma, MO, USA) which is a NF-κB inhibitor, for 1 hour. Then cells were stimulated with LPS (100 ng/ml) for 4 h.The supernatant samples of chondrocytes were collected for the cytokines analysis.

### Transfection

MiR-210 mimic, negative mimic, DR6 siRNA and control siRNA were obtained from Genepharma (Shanghai, China). For transfection, 0.4 nmol mimic or siRNA were mixed with 15 μl Geneporter 2 Transfection Reagent (GTS, San Diego) and then transfected into chondrocyte cells. After 6 h, the supernatant was replaced with fresh medium and cultured for another 48 h.

### Real-time PCR

Total RNA of articular cartilage samples and primary chondrocyte cells was extracted using Trizol reagent (Invitrogen, CA, USA) and reverse transcribed. SYBR Green Gene Expression Assay (Qiagen, Valencia, CA) was used for DR6 and β-actin expression. The PCR primers specific for DR6 were 5′-ACAGAAGGCCTCGAATCTCA-3′ (sense) and 5′-TGCATTCTCGGTCAGTCAAG-3′ (anti-sense); and those for β-actin were 5′-CAACTTGATGTATGAAGGCTTTGGT-3′ (forward) and 5′-ACTTTTATTGGTCTCAAGTCAGTGTACAG-3′ (reverse). TaqMan microRNA assay kit (Applied Biosystems, Foster City, CA) was used for miR-210 analysis. U6 ribosomal RNA was used as the internal control. The primers for U6 snRNA were 5′-GCTTCGGCAGCACATATACTAAAAT-3′, and 5′-CGCTTCACGAATTTGCGTGTCAT-3′. The reverse transcription primer for miR-210 was 5′-GTCGTATCCAGTGCGTGTCGTGGAGTCGGCAATTGCACTGGATACGACTCAGCC-3′. The quantitative RT-PCR primers for miR-210 were 5′-ATGCCTGTGCGTGTGA-3′ and 5′-GTGCGTGTCGTGGAGTC-3′.

### ELISA

Inflammatory cytokines, especially IL-1β, IL-6, TNF-α are thought to mediate the progression of OA^20^. Levels of these cytokines in SF and supernatant samples were detected using ELISA kits (R&D Systems, Minneapolis, MN, USA) according to the manufacturer’s instructions.

### MTT assay

The cell viability of chondrocytes was assessed by MTT assay (MTT kit; Sigma, MO, USA). Cells were grown on 96-well plates at a density of 2 × 10^5^ cells per square centimeter. MTT (0.5 mg/ml) was added to the cells and incubated for 4 h at 37 °C. The supernatant was removed, and the crystals were dissolved in 100 μL DMSO. Absorbance at 570 nm was measured using a Bio-Rad microplate reader (CA, USA). Six parallel wells were set for each group.

### Flow cytometry assay

Cell apoptosis was assayed by flow cytometry using the Annexin V-FITC Apoptosis Detection Kit (Sigma, MO, USA). Briefly, cells were washed twice with PBS and then resuspended in binding buffer. Then cells were incubated with Annexin V-FITC for 10 min at room temperature in the dark. Finally, ropidium iodide was added at a final concentration of 1 mg/L. Stained cells were analyzed using a FACScalibur (Becton Dickinson, Mountain View, CA, USA).

### Western blot analysis

Chondrocyte cells were lysed with M-PER Protein Extraction Reagent (Pierce, Rockford, IL) supplemented with a protease inhibitor. Total cellular protein concentration was determined using Bradford assay kit (Bio-Rad, Hercules, CA, USA) and separated in 10% SDS-PAGE and blotted onto polyvinylidene difluoride (PVDF) membrane. After blocking for 4 h in 5% skim milk, the membrane was incubated with 1:1000 dilution of primary antibodies against DR6, p65, IκBα, β-actin (Santa Cruz Biotechnology, Santa Cruz, CA) at 4 °C overnight. Membranes were rinsed and incubated for 1 h with the corresponding peroxidase-conjugated secondary antibodies. Chemiluminescent detection was performed using an ECL kit (Pierce Chemical, Rockford, IL, USA) and Bio-Rad ChemiDoc MP Imaging System. Gray value of the bands was analyzed by Image J2x software.

### Luciferase activity assay

A segment of the 3′-UTR of DR6 was cloned into pMiR-Report (Ambion Inc., Austin, TX, USA). A mutated 3′-UTR of DR6 was introduced into the potential miR-210 binding site by the two-step PCR approach. The reporter vectors containing the wide type or mutant of *DR6* 3′-UTR and miR-210 mimic were cotransfected into 293T cells (ATCC, Manassas, VA, USA). After 48 h, luciferase activity was measured with a dual-luciferase reporter assay system (Promega, WI, USA).

### Establishment of rat osteoarthritis model

Twenty rats were randomly divided into four groups: normal control group (NC; n = 5), sham surgery group (SS; n = 5), OA model group (OA; n = 5), and OA model with the treatment of miR-210-expressing lentivirus (OA+miR-210; n = 5). The rat model of OA was built by anterior cruciate ligament transection (ACLT) in the right knees as described previously^29^. All rats were anesthetized by intraperitoneal injection of 7 mg/kg xylazine (Rompun; Bayer, Istanbul, Turkey) and 60 mg/kg ketamine hydrochloride (Ketalar; Parke-Davis, Istanbul, Turkey). Right knees of the rats were sterilized with polyvinyl iodine (Betadine, Eczacibasi, Turkey) and a parapatellar skin incision was made on the medial side of joint. In order to expand the operation view, an incision was performed on the medial side of patellar tendon and patella was dislocated laterally with the leg. Anterior cruciate ligament was transected using a #11 surgical blade. A positive anterior drawer test was carried out to ensure complete transection of the ligament. The medial retinaculum was repaired and skin was closed separately. All operative procedures were performed using a loop magnification. The rats in the SS group underwent anaesthesia, and surgical incision in the joint capsule but without ACLT. MiR-210-expressing lentivirus (Hanheng Bio-Tech Co., Ltd., Shang hai, China) was injected into articular cavity of the rats in OA+miR-210 group. For post-operative analgesia, 0.02 mg/kg fentanyl citrate (Fentanyl; Abbott, Chicago, Illinois) was administered subcutaneously twice daily for 3 days after surgery. The same volume of sterile saline was injected in the control group at the same time.

### Samples collection

Five rats in each group were sacrificed by lethal anesthetic overdose on the 20th day after the operation. The articular cartilages of medial tibial plateau were collected and stored at −80 °C for further expression analysis of miR-210, DR6, p65 and IκBα.

SF samples of the rats were collected and processed as described previously^30^. The collected SF was diluted with 2 ml of sterile 0.9% NaCl and then was passed through a 1.2 μm filter. 10% (v/v) proteases and phospholipases inhibitors were added. The mixture was centrifuged at 16,000 *g* for 45 min at room temperature (RT) and the supernatant was collected and frozen at −80 °C for cytokines analysis.

### Data analysis

The experiments were carried out for three times, except for the model establishment. All data are expressed as means ± standard deviation (SD). Statistical analysis was performed using an unpaired T test (Fig. 1) and a one-way ANOVA (Figs 2, 3, 4, 5) by GraphPad Prism 6 software (GraphPad Prism Software, Inc.). *P *< 0.05 was considered as significant difference.

## Additional Information

**How to cite this article**: Zhang, D. *et al*. MiR-210 inhibits NF-κB signaling pathway by targeting DR6 in osteoarthritis. *Sci. Rep*. **5**, 12775; doi: 10.1038/srep12775 (2015).
